# Supplementary material for: Eco-Friendly Synthesis of Quinazoline Derivatives Through Visible Light-Driven Photocatalysis Using Curcumin-Sensitized Titanium Dioxide
Source: Materials (Basel). 2024 Dec 20;17(24):6235. doi: 10.3390/ma17246235 (PMC11676707; doi:10.3390/ma17246235)

Supplementary materials

Eco-Friendly Synthesis of Quinazoline Derivatives through Visible Light-Driven Photocatalysis Using Curcumin-Sensitized Titanium Dioxide

Mshari A. Alotaibi[a]*, Abdulrahman I. Alharthi[a], Talal. F.Qahtan[b], Md. Afroz Bakht[a]

[a] Department of Chemistry, College of Science and Humanities in Al-Kharj, Prince Sattam Bin Abdulaziz University, P.O. Box 173, Al-Kharj, 11942, Saudi Arabia

[b] Department of Physics, College of Science and Humanities in Al-Kharj, Prince Sattam Bin Abdulaziz University, P.O. Box 173, Al-Kharj, 11942, Saudi Arabia

*Correspondence: alosaimi@psau.edu.sa

NMR data

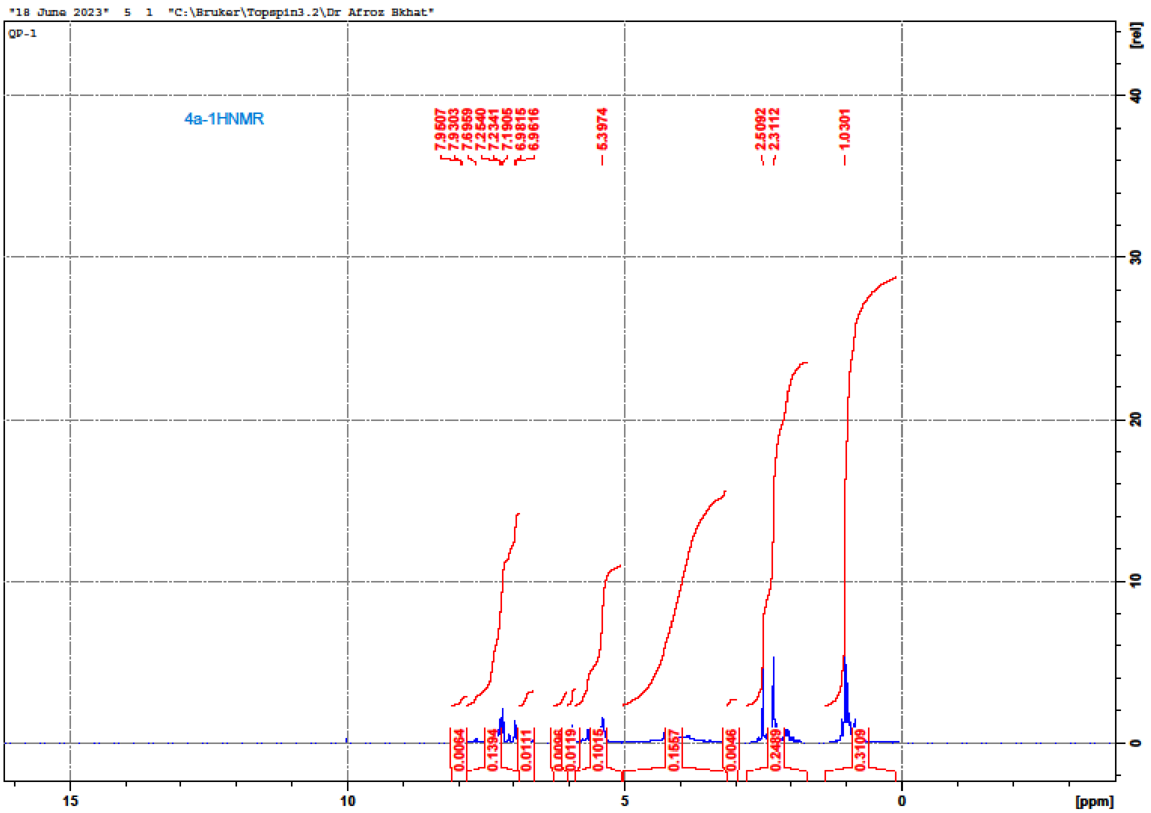

Supplementary materials

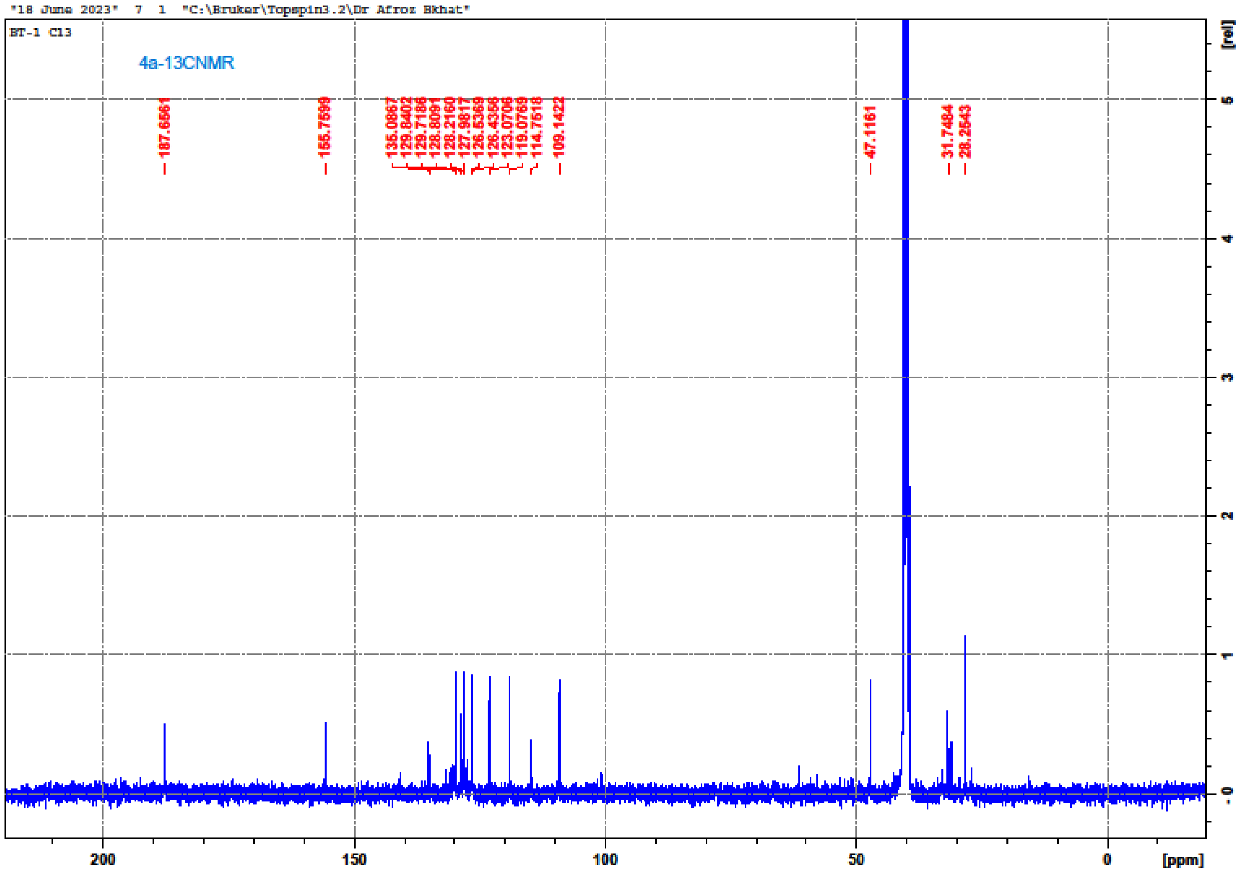

Supplementary materials

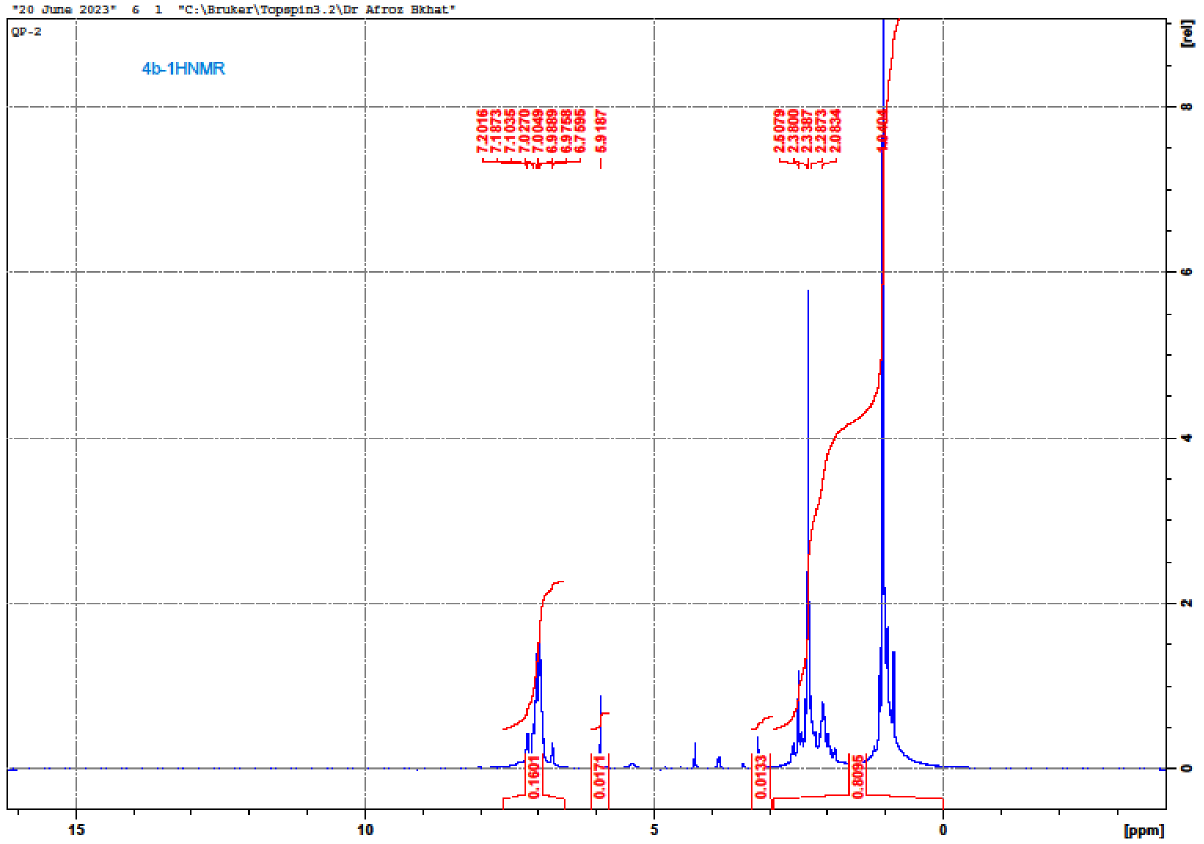

Supplementary materials

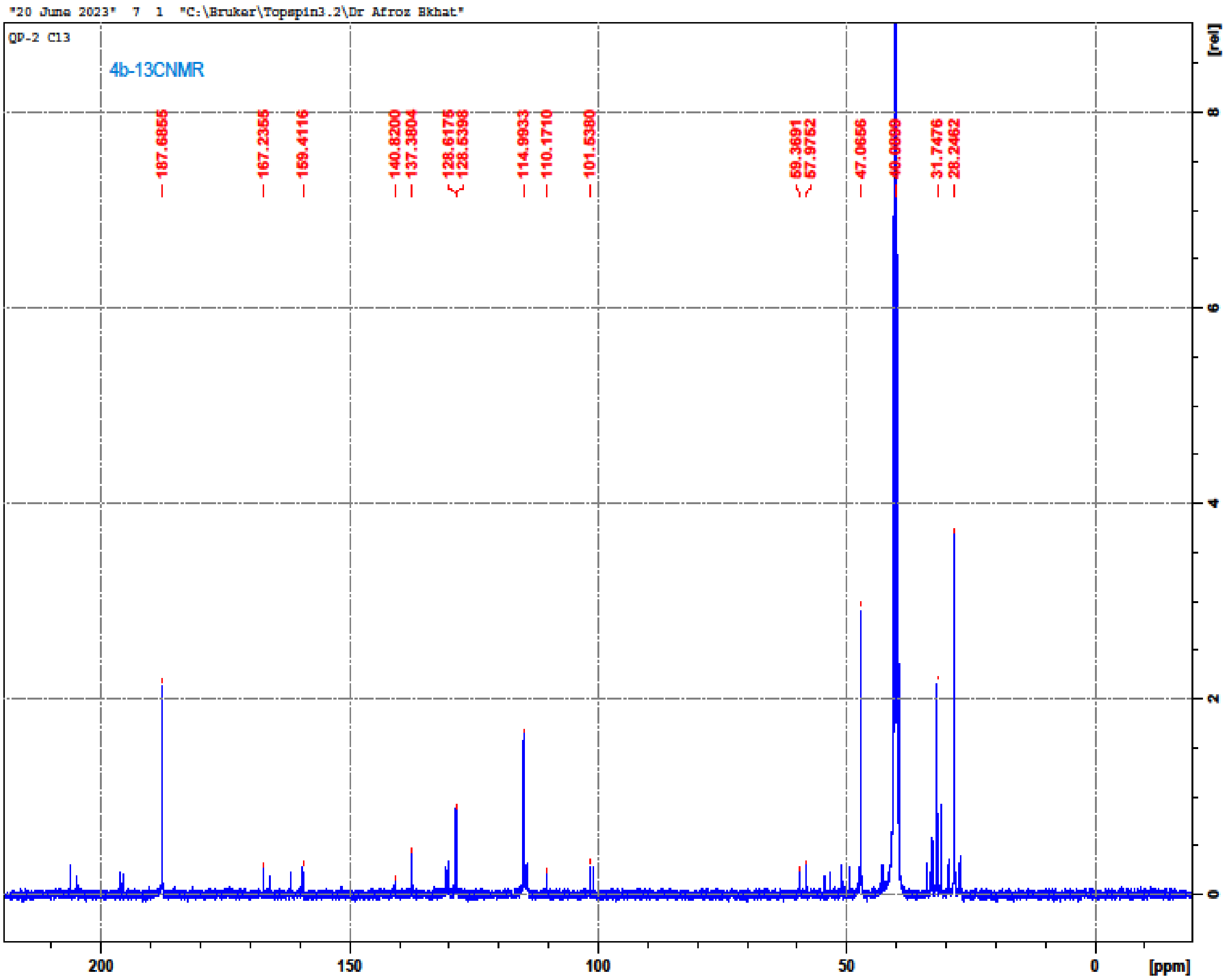

Supplementary materials

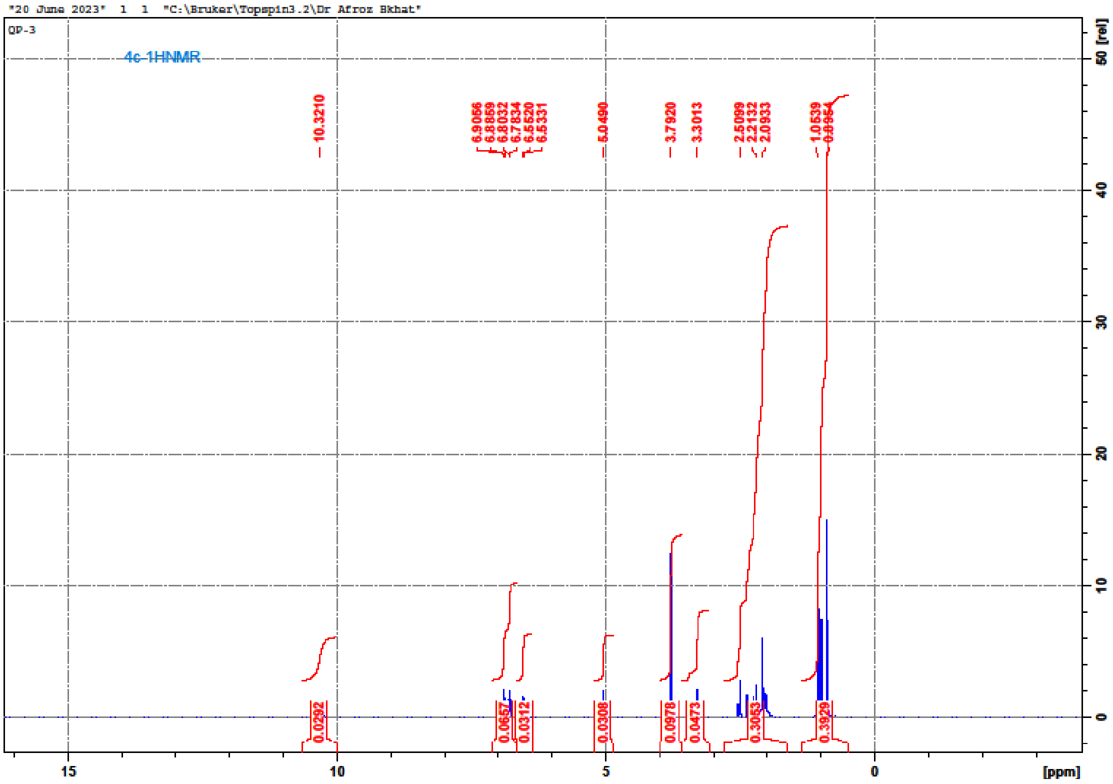

Supplementary materials

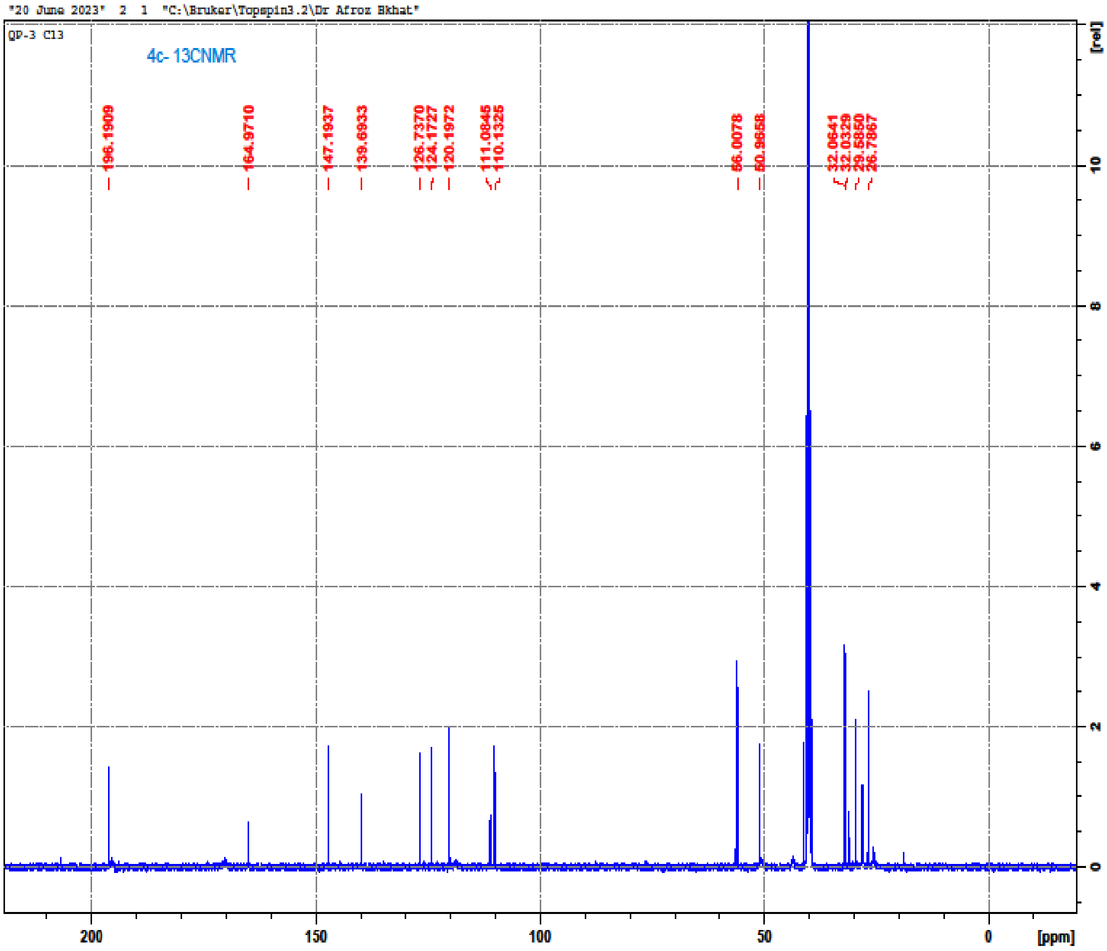

Supplementary materials

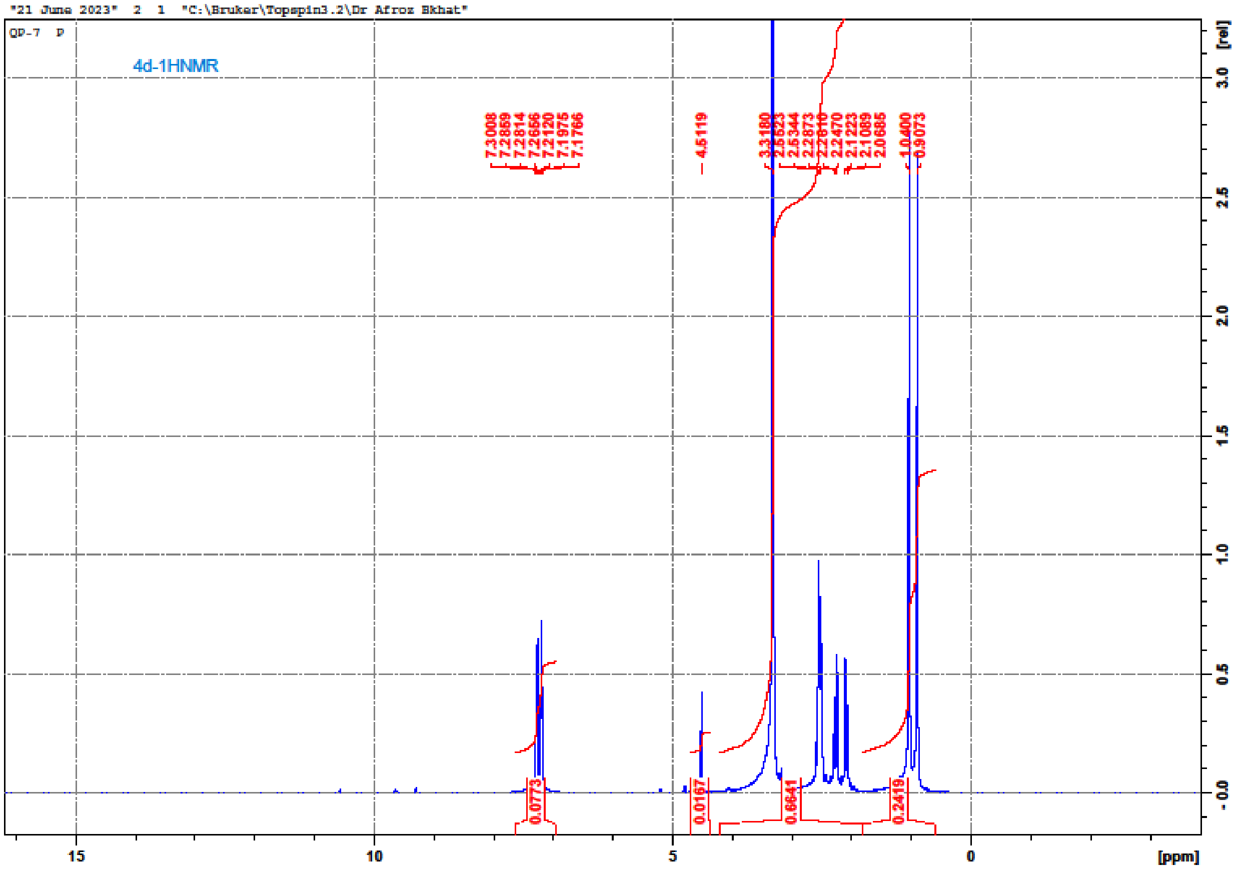

Supplementary materials

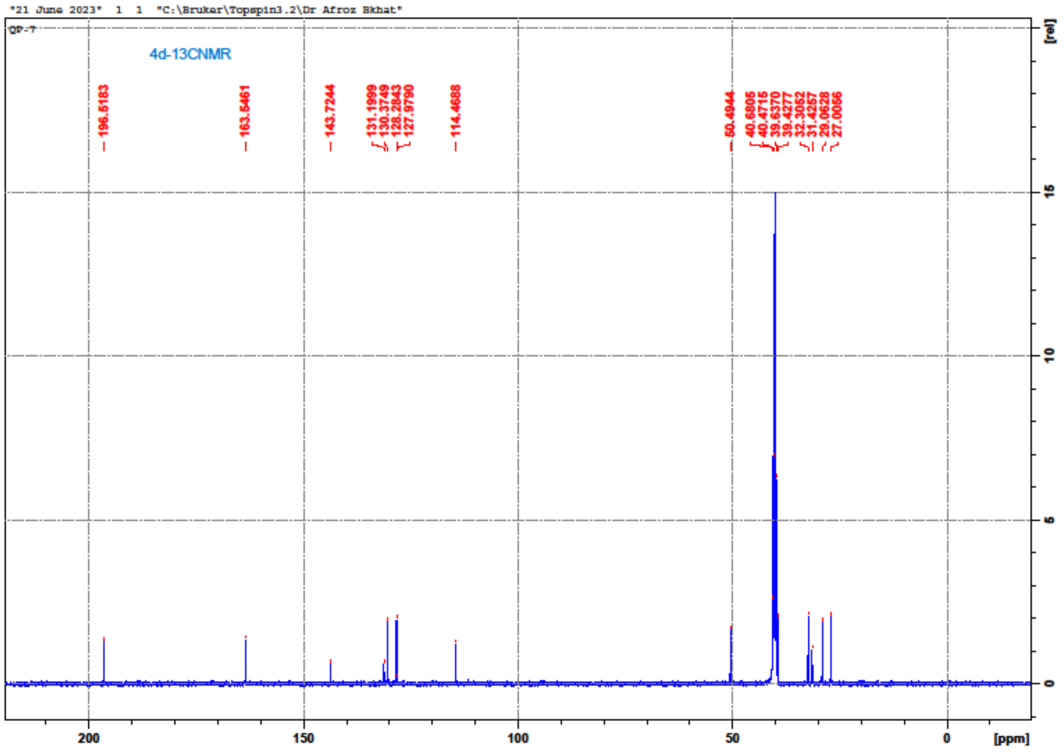

Supplementary materials

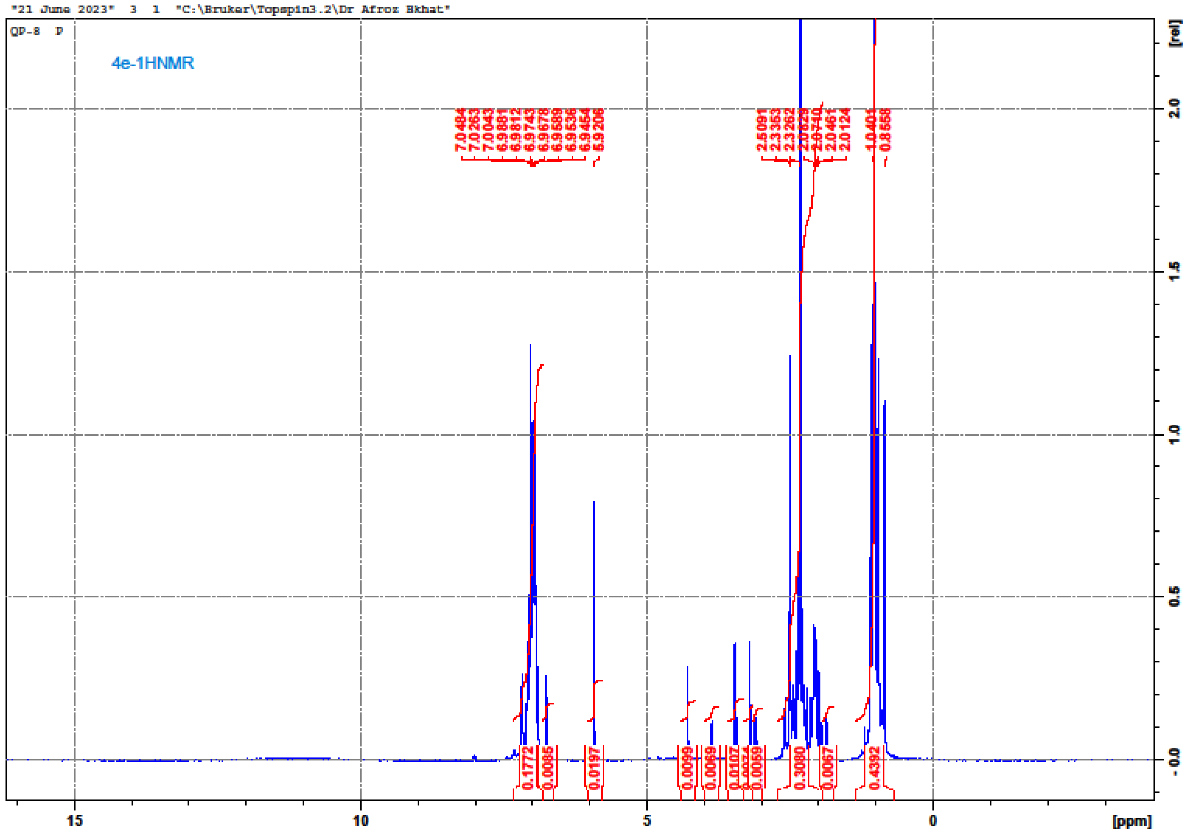

Supplementary materials

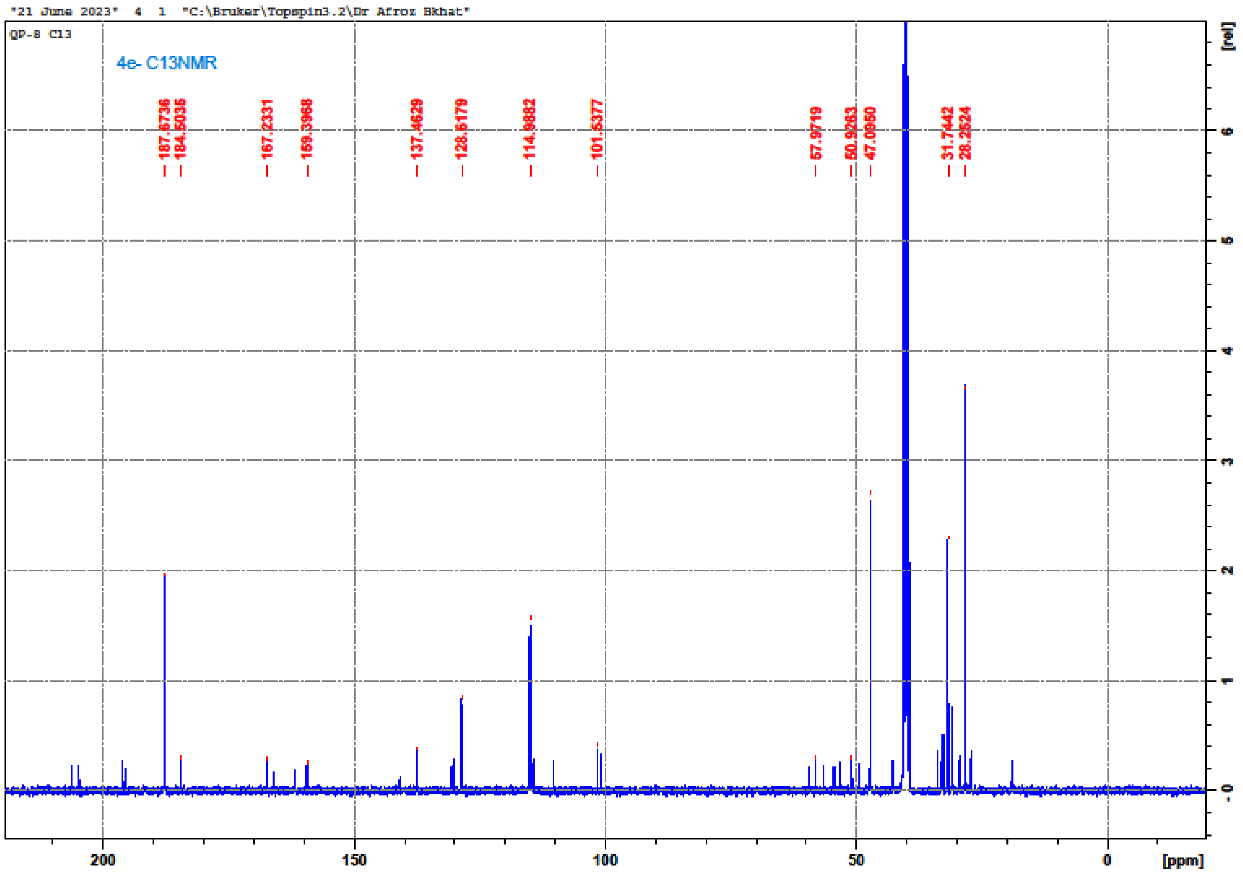

Supplementary materials

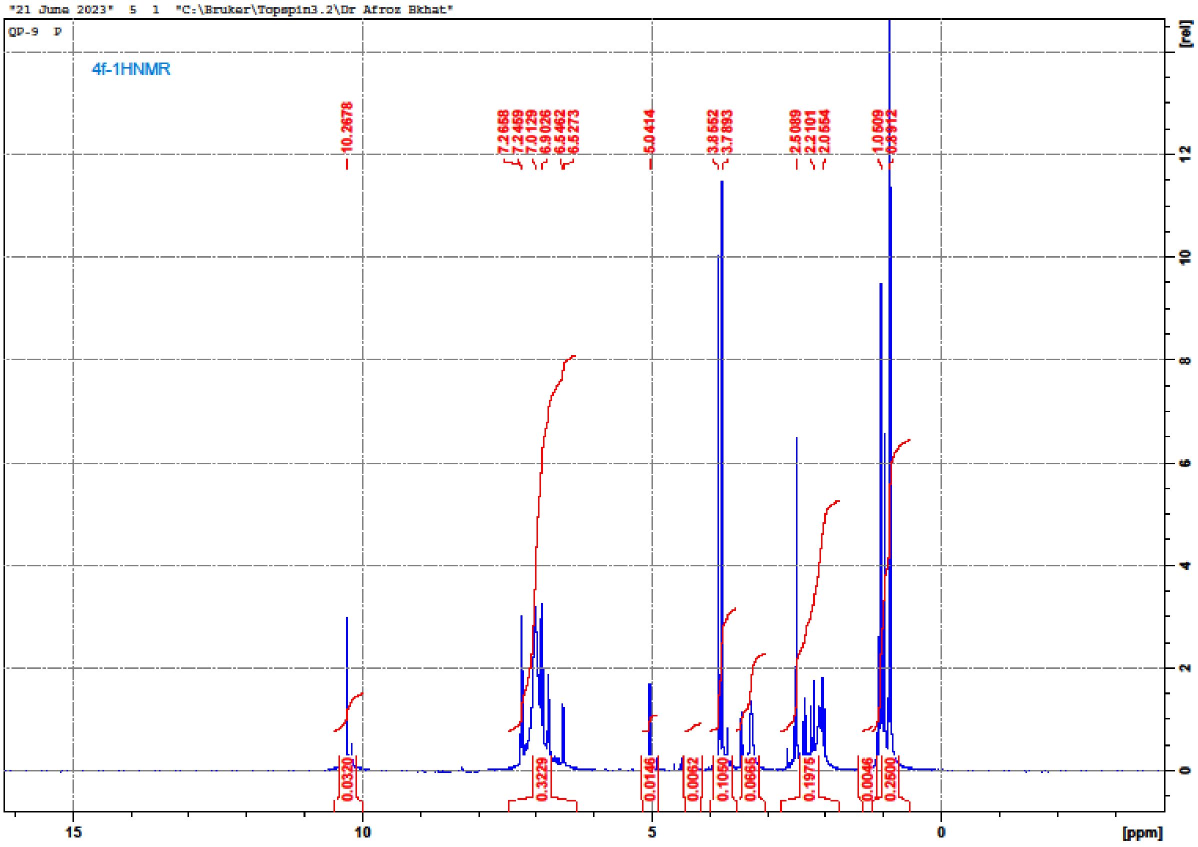

Supplementary materials

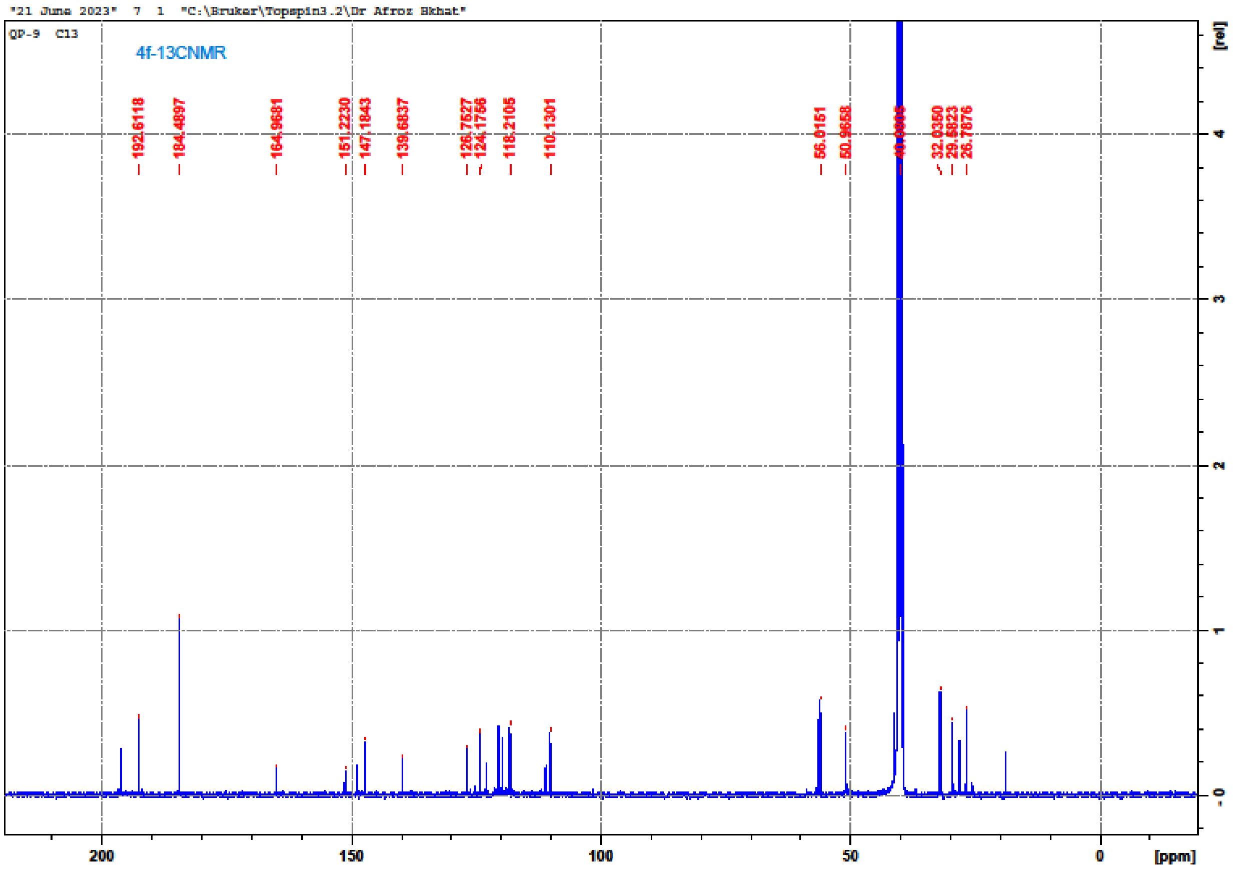

Supplement: Supplementary file 1 [file materials-17-06235-s001.zip › materials-3298802-supplementary.pdf]
